# Fishery Discards: Factors Affecting Their Variability within a Demersal Trawl Fishery

**DOI:** 10.1371/journal.pone.0036409

**Published:** 2012-04-30

**Authors:** Jordan Feekings, Valerio Bartolino, Niels Madsen, Tom Catchpole

**Affiliations:** 1 National Institute of Aquatic Resources, Technical University of Denmark (DTU-Aqua), Hirtshals, Denmark; 2 Department of Aquatic Resources, Swedish University of Agricultural Sciences, Lysekil, Sweden; 3 Department of Earth Sciences, University of Gothenburg, Gothenburg, Sweden; 4 Centre for Environment, Fisheries and Aquaculture Science, Lowestoft, United Kingdom; Aristotle University of Thessaloniki, Greece

## Abstract

Discards represent one of the most important issues within current commercial fishing. It occurs for a range of reasons and is influenced by an even more complex array of factors. We address this issue by examining the data collected within the Danish discard observer program and describe the factors that influence discarding within the Danish Kattegat demersal fleet over the period 1997 to 2008. Generalised additive models were used to assess how discards of the 3 main target species, Norway lobster, cod and plaice, and their subcomponents (under and over minimum landings size) are influenced by important factors and their potential relevance to management. Our results show that discards are influenced by a range of different factors that are different for each species and portion of discards. We argue that knowledge about the factors influential to discarding and their use in relation to potential mitigation measures are essential for future fisheries management strategies.

## Introduction

Discards refers to the organisms of both commercial and non-commercial value that are caught during commercial fishing operations and returned to the sea, often dead or dying [1]. The practice of discarding occurs for a range of reasons, including, individuals caught are under the minimum landing size, species have a low or no market value, catch is damaged or is high-graded (i.e., lower valued individuals are discarded to maximize profits), or the species quota is reached. Three fundamental causes are responsible for the high level of discarding in European Union (EU) fisheries, namely the use of unselective fishing techniques, the failure to reduce fishing effort, and biological and environmental factors affecting the distribution of species [2]. A multitude of other factors also affect the practice of discarding, such as complex social [3], technical [4], [5], economical [6]–[8], and legislative [9] reasons. In addition, the effect and relative importance of these factors will vary for different species, vessels, metiers (*fishing operations characterised by the same fishing gear and catch composition*) and fleets, and will fluctuate over time [10] and space [9]. As a further source of variation there is the individual choice by fishermen as to which part of the catch to retain and which to discard [3], [10].

Furthermore, discarding has wider implications whereby ecosystem functioning and its biodiversity are negatively affected [11]. There are indications that discarding has altered the ecosystem functioning of some seabird communities [12], [13] and has negative effects on charismatic and endangered species [14]. The European Commission (EC) considers discarding to be negative, both in terms of ecosystem functioning and economic viability, and is committed to eradicating the problem [11]. The Common Fisheries Policy (CFP) reform, to be introduced in 2013, is set to eliminate the problem of discards through the introduction of a discard ban [15].

The most complex discard problems are found in mixed-species demersal trawl fisheries, and are responsible for most of the discards [2], [3]. In the Kattegat, the demersal trawl fishery, the focus of this study, is the dominant gear type, accounting for approximately 80% of all fishing effort [16]. The fishery has been faced with regulatory measures for the recovery of the Kattegat cod (*Gadus morhua*), which has largely been unsuccessful so far [17]. The small mesh sizes currently and previously employed in the Kattegat are used to retain Norway lobster (*Nephrops norvegicus*) and sole (*Solea solea*). This may lead to high discard rates of juvenile round and flatfish species [17]–[19]. A similar occurrence has been observed in the North Sea beam trawl fishery for sole where high discarding of plaice (*Pleuronectes platessa*) occurs [20]–[22].

The present literature on discards has mainly been descriptive, with a focus on understanding discard rates of specific species [23], estimating the amount or proportion of total catch discarded from particular fisheries [9], [24], species and length compositions of discards [6], [25], [26], as well as global discard estimates [14], [27]. While these studies help provide a better insight into the discarding problem there is a lack of quantitative studies regarding discarding behaviours [4]. Therefore, further knowledge of the factors that influence discard rates is needed. Studies of such nature can help to gain an insight into the factors influencing the discarding process, and to predict future catches and discards [28]. The use of modelling approaches to discard data provides the possibility to further disentangle the effects of different drivers [29] and offers important insights into the potential effectiveness of technological and area/time management measures for reducing fishery discards [30]. Modelling approaches can also prove useful in the process of systematically reducing by-catch in multispecies fisheries, complementing classic mesh retention experiments as aids to the development of strategies to reduce discard levels [30], and providing support for mesh size increases. Modelling discards is an area that has received little research effort, but requires research [4], [29], [31].

Past studies that have modelled the relationship between explanatory variables and discards have focused on discards as a whole [4], [28], [30], [31] without the consideration that different portions are discarded for different reasons. For example, discarding of individuals under minimum landing size (<MLS) mainly occurs as a result of the MLS and juvenile abundance, while larger individuals, those above minimum landing size (≥MLS), are discarded for a variety of different reasons, including landing composition regulations, no quota, market forces, damaged, or the species has a low or no economic value. There are also many environmental [4] and vessel/gear specific parameters that have also been suggested to influence discard rates [17]–[19]. These will act differently on different portions of discards and may only influence one portion or species.

The high number of potentially influential factors stems from the complexity of the social, economic, management, and environmental forces acting on the system. If a theory of discards is to emerge, all potentially relevant factors should be considered [4]. This study elaborates on the existing knowledge by considering a larger number of factors that can potentially influence the variability of discards < and ≥MLS. We apply a Generalised Additive Model (GAM) using discard data from the Danish discard observer programme for the demersal trawl fishery in the Kattegat to identify the driving factors that influence discarding practices and those that could potentially be important for the development of management strategies.

## Materials and Methods

### Discard data

Since 1995 Denmark has collected data on catches and discards with the aim of sampling all demersal fisheries except the ones with very limited fishing effort and discard. In 2002 the EU identified the need to describe and quantify discards as part of the European Data Directive (1639/2001 and 199/2008). The data collected is stratified with regards to ICES area, quarter, and discard pattern of the relevant fisheries (e.g. fisheries with low discards are seldom sampled). Participation in the discard sampling programme is opportunistic, i.e. permission by the skipper is required, and as the observer has no relation to the control unit, the fishing practice is assumed to be unaffected by the observers presence. In order for the sampling programme to be representative of the fisheries in question, vessels of all sizes are sampled from all the main fishing harbours during the entire period of activity of a given fishery. Biological information (i.e., lengths, weights and otolith samples) are collected from the catch, together with vessel, gear, geographical position and environmental attributes (depth, bottom type).

For each observed haul, an estimate of the total catch weight is made by the fishermen and the observer in collaboration. The total catch is then sorted into the retained and discarded components by the commercial fishermen. The total weights of each individual species retained are recorded. If the abundance of a species is small, total numbers and lengths are recorded, otherwise a subsample is taken, numbers and lengths recorded and raised accordingly. The total weight of the discarded portion is approximated, a subsample taken, and then sorted by the observer into species. Total weights and numbers of each discarded species in the subsample are determined and raised based on the total approximated discarded weight.

**Figure 1:**
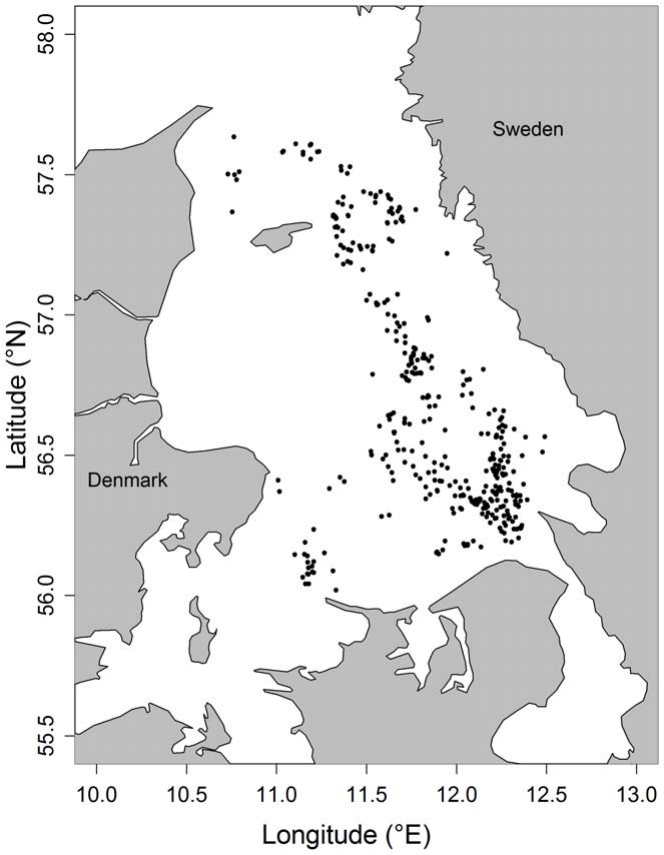
Map of the study area with the location of discards observer hauls used in the analysis. Locations represent the mid-way point of each haul.

**Figure 2:**
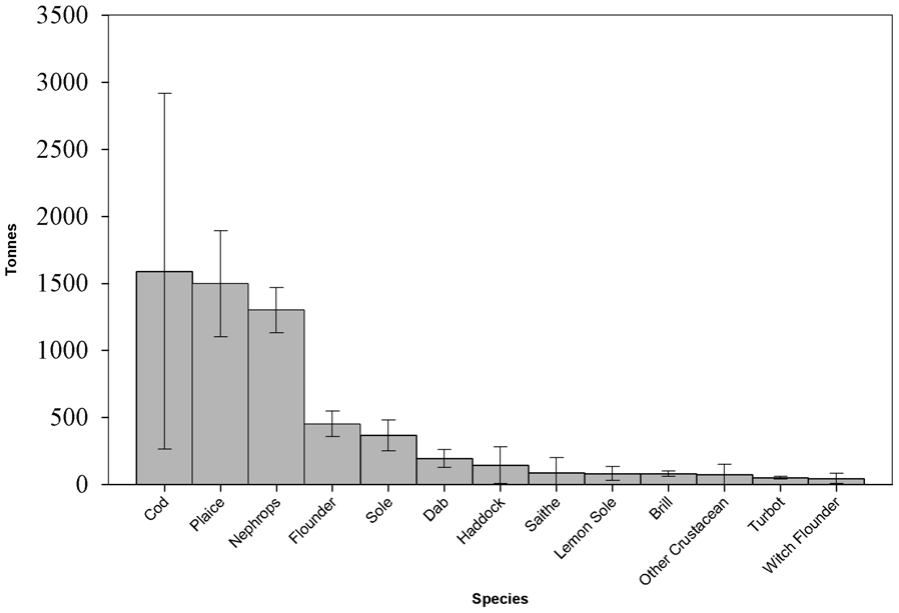
The official Danish landing statistics (www.fvm.dk) for the Kattegat: 1997 to 2008. Species are ranked according to their relative importance by weight. Average value in thousands with standard deviation for the 12 years investigated.

Between 1997 and 2008, 189 trips and 370 hauls were sampled within the Danish demersal bottom trawl fleet active within the Kattegat (Figure 1). The fishery was classified into mesh size categories (full mesh opening); 70–89 mm, 90–99 mm, and 100–120 mm. This was in order to understand the effect of mesh size while ensuring a reasonable number of observations within each size category. Initial analysis on the relative importance, in terms of landings (by weight), revealed that cod, plaice and Norway lobster were the 3 main commercial species targeted by the Danish demersal trawl fleet in the Kattegat (Figure 2). Sole, while caught in relatively low numbers, was the second most important species economically. Due to the large difference in landings from other species and the fact that Norway lobster is one reason why small mesh sizes are used, these 3 species are the main focus of this study. The Kattegat demersal trawl fishery is a mixed fishery and has the most comprehensive discard sampling in relation to other fisheries in the area, namely Danish seines and static gears (mainly gillnets).

### Regulations

Regulations throughout the 12 year study period changed considerably. Mesh sizes increased and square mesh panels to improve selectivity became an option in the legislation. In 2005 the minimum diamond-mesh size in codends used in the Kattegat increased from 70 to 90 mm, unless a sorting grid is used in combination with a 70-mm square mesh codend (EC Council Reg. 27/2005). A 120-mm square mesh panel inserted in a 90-mm codend was introduced as a voluntary option in the legislation from 2005 [19]. From our knowledge, uptake of the window was very minimal by the industry in the Kattegat. A previous study found no significant improvement in selectivity for the three species investigated here [18]. Therefore, mesh sizes categories are grouped regardless of whether or not selectivity windows were present. Quotas were split into fortnightly rations which were continuously adjusted to the amount of quota left. In 2007 individual transferable vessel quotas were implemented in the Danish demersal fishery whereby a vessel is allocated an annual quota for each species. The cod quota decreased tenfold over the 12 year study period; from 5170 tonnes in 1997 to 465 tonnes in 2008. The quotas for plaice and Norway lobster both remained relatively stable during the same period; the plaice quota fluctuated around 2200 tonnes±500 tonnes; while the Norway lobster quota increased marginally, from around 3500 tonnes in 1997 to around 4000 tonnes in 2008. The MLS for the three species remained unchanged throughout the study period (i.e., cod 35 cm, plaice 27 cm, Norway lobster 13 cm (4 cm) total length/carapace.

### Statistical analysis

To account for the unbalanced sampling design between explanatory variables, and describe the main spatial distribution changes over time, generalised additive models (i.e. GAMs, [32] were used. A quasi-Poisson distribution (log-link) was used because the data are counts without an upper limit, and overdispersed (i.e. variance exceeds the mean or contain a large number of zero observations). The quasi-likelihood approach assumes that the scale parameter Φ of the distribution is unknown, which makes it more suitable for over-dispersed data than the classical Poisson distribution [33]. The variance of a quasi-Poisson model is a linear function of the mean [34]. Rather than use density of discards (numbers per hour) as a response variable, we chose to model numbers discarded per haul with the use of an offset variable (haul duration). The advantages of the offset approach compared to analysing densities are that the fitted values are always positive, the confidence intervals around the fitted values do not contain negative values, and we allow for heterogeneity within the context of a Poisson distribution [35]. Of further interest was what effect vessels had on discards, although we are not interested in knowing the exact nature of the vessel effect. Therefore we include vessel as a random effect. Here we assume that the variation around the intercept, for each vessel, is normally distributed with a certain variance.

A large number of potential variables were considered for each of the models and through exploratory analysis and a stepwise deduction using a priori knowledge a total of 11 variables were included in the analysis (Table 1). Some variables were only available or specific for a species or a subcomponent and therefore not included in all models, i.e. Juvenile abundance was only available for cod <MLS while quota utilisation was available for the cod and plaice models ≥MLS. To simplify the interpretation of the results, the maximum degrees of freedom (measured as number of knots k) allowed to the smoothing functions were limited for the variables total catch weight, juvenile abundance, vessel power and depth (k = 4). The full model was formulated as follows:
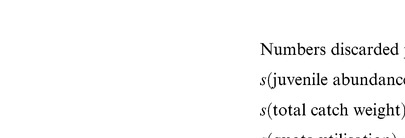
where *β* is an overall intercept, *s* is an isotropic smoothing function (thin-plate regression spline), and *ε* is an error term.

**Table 1:** Summary of variables included in Generalised Additive Models of factors influencing discards.

Variable	Description	Comments
**Year**	Year haul was sampled	1997–2008
**Quarter**	Quarter haul was sampled	
**Depth**	Mean fishing depth of haul	In meters
**Longitude, Latitude**	Mean Longitude/Latitude of haul	In decimal degrees
**Mesh size category**	Codend mesh size of trawl	70–89 mm, 90–99 mm, 100–120 mm
**Juvenile abundance**	Abundance of age 1 and 2 individuals per quarter^1^	
**Total catch weight**	Total catch of all species	in kilograms
**Quota utilisation**	Amount of quota left^2^	
**Vessel power**	Engine size of vessel	Used as a proxy for the size of the trawl
**Haul duration**	Haul duration in hours	Used as an offset term
**Vessel**	Unique code for each vessel	Used as a random effect

Based on data collected as part of the discard sampling programme 1997–2008.

1 Juvenile abundance was only available for cod.

2 Quota utilisation was only assessed for cod and plaice. The quota for Norway lobster is not restrictive and therefore does not affect discards.

The effect of juvenile abundance on discard rates was only tested for cod discards <MLS. No stock assessment is carried out for Norway lobster, and the plaice assessment is for Kattegat and Skagerrak combined, hence the variable year was used instead to account for the year effect. The variable “juvenile abundance” per quarter was calculated by applying a simple exponential decay function based on the relative number of individuals of age 1 and 2 caught during Baltic sea International Trawl Surveys (BITS) undertaken in the first quarter of each year. Natural mortality (M) and Fishing mortality (F) were taken from the official assessment (ICES, 2011) where we assume that M is constant during the year and F increases linearly during the year (this is to account for the growth, and subsequent increase in retention by the fishing gear, of an individual throughout the year). One year old cod are approximately 18 cm in length, which corresponds approximately to the L50 (length at which fifty percent of the fish are retained in the cod-end) of the smallest mesh size (70 mm) used [17]. It is also assumed that fish of age 2 in quarters 3 and 4 have length ≥MLS and are thus excluded from the index of juvenile abundance.

All potentially important covariates were included in the initial model where the least significant covariates were removed one at a time until all covariates were significant (P<0.05). The final models are then reduced versions of these full models. The analyses were performed using R software, a statistical environment for computation and graphics (http://www.r-project.org), and the R package ‘mgcv’ [36].

## Results

A summary of the discard data is presented in Table 2. A total of 370 demersal hauls were analysed over the period 1997 to 2008 in the Kattegat. All models considered are presented in Table S1. The final models together with each covariates degrees of freedom, significance level and the deviance explained by the model are presented in Table 3. The final models explained between 49 and 83% of the deviance. Visual analysis of the model residuals revealed no violation from any of the model assumptions (i.e., normality and homogeneity of variance). The residuals were also inspected for spatial autocorrelation.

**Table 2:** Summary of discard data collected onboard demersal trawls in the Kattegat for the three mesh size categories.

	Mesh size categories		
	70–89 mm	90–99 mm	100–120 mm
**Years (no.)**	8	9	11
**Vessels (no.)**	19	12	9
**Horse power (kW)**	331.8 (104.6)	367.1 (119.7)	388.0 (108.4)
**Haul duration (hrs)**	6.5 (1.8)	6.2 (1.7)	5.2 (1.6)
**Hauls (no.)**	168	132	70
**Avg. catch weight (kg)**	625.8 (393.2)	576.5 (307)	953.9 (713.2)
**Avg. discard cod <MLS (no./hour)**	31.2 (44.3)	14.9 (18.2)	13.8 (18.4)
**Avg. discard cod ≥MLS (no./hour)**	0.5 (1.2)	0.9 (3.4)	1.9 (3.6)
**Avg. discard plaice <MLS (no./hour)**	64.7 (104.1)	43.0 (56.4)	32.7 (54.7)
**Avg. discard plaice ≥MLS (no./hour)**	1.3 (4.1)	1.7 (6.1)	14.2 (25.7)
**Avg. discard Norway lobster <MLS (no./hour)**	450.5 (605.44)	273.6 (385.3)	6.6 (39.1)
**Avg. discard Norway lobster ≥MLS (no./hour)**	17.0 (75.3)	10.5 (20.2)	0.2 (1.2)

Standard deviations are in brackets.

**Table 3:** Final models together with each covariates degrees of freedom, significance level and the deviance explained by the model.

	Predictors											
model	α	βmesh	βquarter	s(Yr)	s(catch)	s(JuvAb)	s(lon,lat)	s(quota)	s(vessel kW)	s(depth)	s(Vessel)	DEV.EXPL(%)
**cod**												
**<MLS**	−2.97**	70–89 (1.85)**			2.96**	2.98**	22.08**					63.3
		90–99 (1.86)**										
**≥MLS**	−4.15**		Q2 (−0.30)	7.07**	2.82**		19.41**	2.80**				64.5
			Q3 (−2.20)**									
			Q4 (−1.49)*									
**plaice**												
**<MLS (a)**	−1.54**	70–89 (1.20)**		6.96**	2.42**		19.57**					61.8
		90–99 (1.03)**										
**<MLS (b)**	−1.21**		Q2 (0.59)**	5.97**	2.59**		19.59**					61.3
			Q3 (0.69)**									
			Q4 (0.97)**									
**plaice ≥MLS**	−2.30*	70–89 (−2.25)**	Q2 (−1.12)*	6.04**	1.60**		21.81**	2.77**		1.00*	18.46**	81.8
		90–99 (−1.62)**	Q3 (−2.32)**									
			Q4 (−1.54)									
**Norway lobster**												
**<MLS**	−2.54**	70–89 (1.38)*	Q2 (1.73)**	7.92**	2.63**		22.21**				20.32**	83
		90–99 (0.91)	Q3 (1.83)**									
			Q4 (1.75)**									
**≥MLS**	−6.07**	70–89 (2.37)*	Q2 (1.50)**	7.65**	2.05**		18.25**		1.32**	1.00*		49.2
		90–99 (1.78)	Q3 (1.91)**									
			Q4 (1.29)*									

Significance levels: 0.001 ‘**’ 0.01 ‘*’, α = intercept, mesh = mesh size category, quarter = quarter of the year, Yr = year, catch = Total catch weight, JuvAb = Juvenile abundance, lon = longitude, lat = latitude, quota = species quota utilisation, vessel kW = vessel power, depth = mean fishing depth, vessel = individual vessel id.

**Figure 3:**
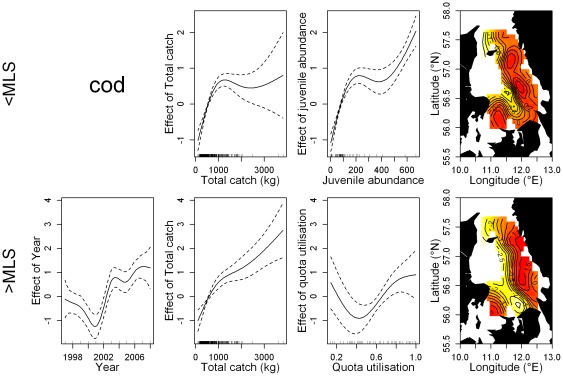
Effect of the significant smoothing functions (solid line) on the discard rate of cod in the Kattegat demersal trawl fishery. Cod <MLS (top row) and ≥MLS (bottom row). Dotted lines represent the 95% confidence limits. Vertical bars along the x-axis indicate observational values. The surface and contour lines describe the effect of 2-d smoothing function on the geographical coordinates.

**Figure 4:**
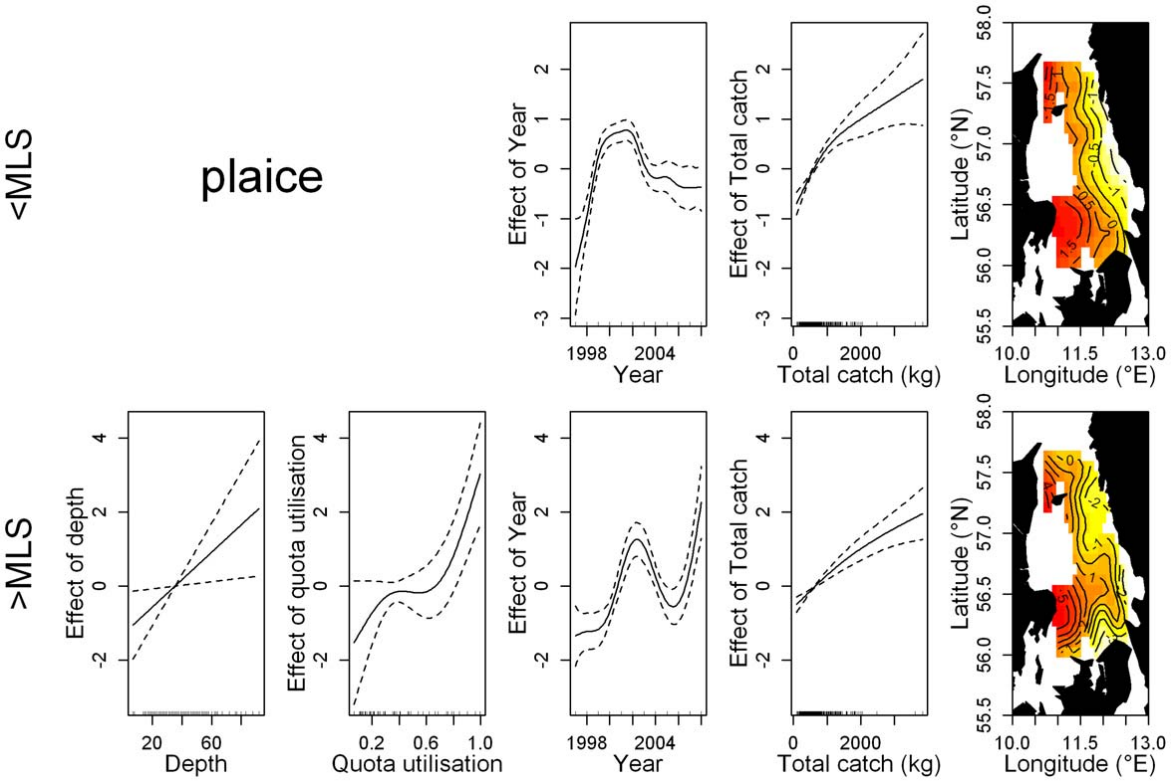
Effect of the significant smoothing functions (solid line) on the discard rate of plaice in the Kattegat demersal trawl fishery. Plaice <MLS (top row) and ≥MLS (bottom row). Dotted lines represent the 95% confidence limits. Vertical bars along the x-axis indicate observational values. The surface and contour lines describe the effect of 2-d smoothing function on the geographical coordinates.

**Figure 5:**
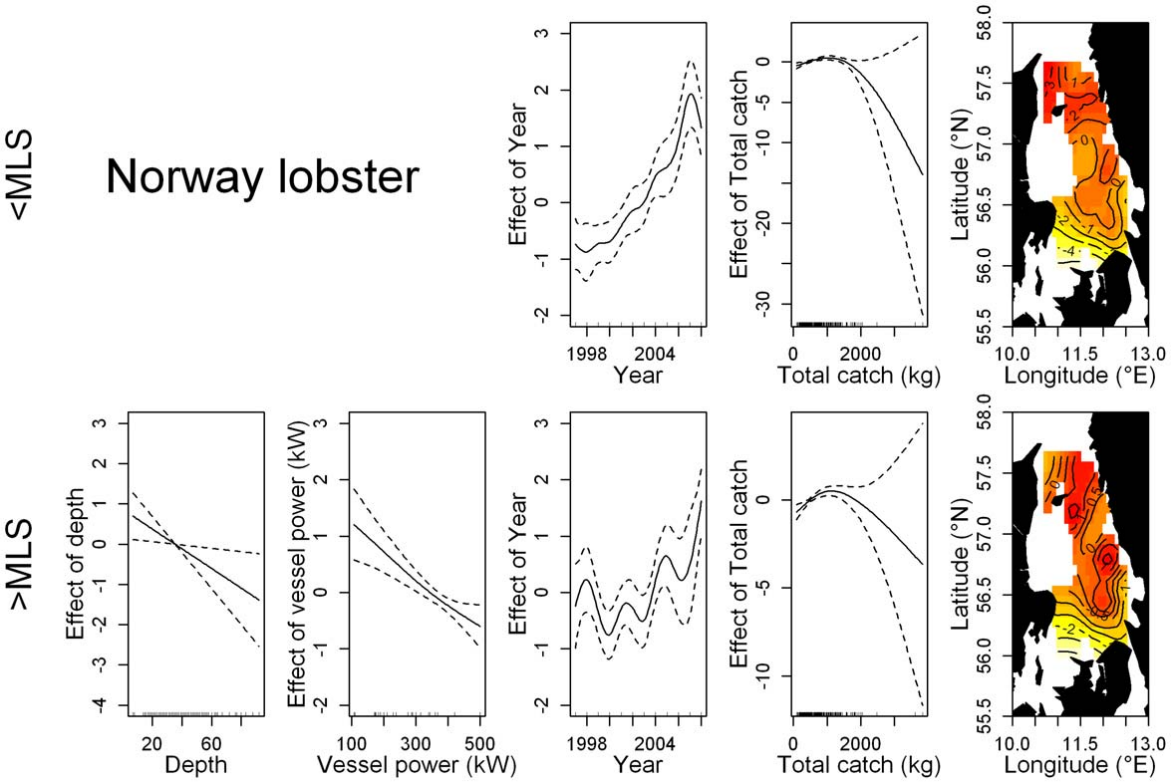
Effect of the significant smoothing functions (solid line) on the discard rate of Norway lobster in the Kattegat demersal trawl fishery. Norway lobster <MLS (top row) and ≥MLS (bottom row). Dotted lines represent the 95% confidence limits. Vertical bars along the x-axis indicate observational values. The surface and contour lines describe the effect of 2-d smoothing function on the geographical coordinates.

The GAMs showed that the relative importance of each variable was different for each species and portion of the discards, with a few similarities (Table 3). In all the final models total catch weight and the interaction between longitude and latitude had a significant effect on numbers discarded. A significant positive relationship between total catch weight and the amount of discards was observed for plaice discards < and ≥MLS and cod discards ≥MLS (Figures 3 & 4). A positive relationship was also observed for cod discards <MLS, however, only for total catch weights up to 1000 kg. For Norway lobster < and ≥MLS, discard numbers tend to decline after a certain point with increasing catch weight, although large uncertainty is associated to large catch estimates (Figure 5). The potential effect of few very large catches (i.e., >3000 kg) has been investigated by removing these observations and refitting the model. The effect of large catches was found to be irrelevant on the dome-shape response of discards rates to the total catch weight.

The effect of the spatial component was consistent for the two portions of the discards within each species, but marked differences were found among the species. Discards of cod were highest in the central eastern part of the Kattegat along the Swedish coastline and in the south western region close to Denmark. Plaice discards on the other hand exhibited a marked longitudinal gradient, with increasing discards westward. The lowest discards of Norway lobster occur in the south with a bimodal increase northward. However, an opposite local effect was observed for the two portions of the Norway lobster discards in the north-west tip of the study area. Here discards <MLS are at their highest while discards ≥MLS exhibit a decreasing trend in a westerly direction. Juvenile abundance had a significant positive effect on the discard rate of cod <MLS (Figure 4; Table 3). Norway lobster discards were highest during the third quarter (Table 3; Figure S1). Plaice discards were highest during the winter months; quarters 4 and 1 for subcomponents < and ≥MLS respectively. Cod discards ≥MLS were highest in the first quarter of the year. Quota utilisation was assessed for cod and plaice discards ≥MLS and found to be highly significant. A positive linear relationship was observed for plaice, while cod discards first declined before increasing as the quota was fished up. Mesh size category was found to have a negative relationship with the amount of discards in all models except for cod discards ≥MLS (Table S1). Depth was non-significant in a majority of the models. For plaice ≥MLS, discarded numbers increase as depth increases while the opposite was observed for Norway lobster discards ≥MLS.

For plaice discards <MLS we end up with two competing final models. When mesh size category and quarter were both included in the model they compete with one another, resulting in non-significant parameters. While when either one was dropped from the model the other one becomes highly significant. Their contributing effects cannot be estimated simultaneously. This is most likely a result of the heterogeneity in the sampling across mesh sizes and quarters.

## Discussion

Knowledge about the reasons why discarding occurs is considered a key element in the progress towards a theory of discarding [4]. We demonstrate that discard rates of the 3 main target species in the Danish Kattegat demersal trawl fishery are influenced by a multivariate and complex range of factors that differs for each species and their subcomponents. Previous studies that have investigated the factors influencing discards did not consider the size composition of the discarded catch, nor distinguish between the reasons that may drive discarding fish of different sizes. Some factors may only be able to influence one subcomponent.

Dealing with discards ≥MLS is a much more problematic task as these are influenced by a range of factors that differ for vessels, fleets, seasons, area and species. Identifying the main influential factors of discards ≥MLS is also much harder. It is difficult to distinguish whether a vessel is discarding marketable fish due to market forces, low or no available quota or the individuals are damaged. Discards ≥MLS are often a result of market or regulatory constraints from the quotas and rations in place. Quotas in the Danish demersal fisheries for years 1997–2006 were split into fortnightly rations which were continuously adjusted to the amount of quota left. As the quotas were fished up the rations were reduced to try and sustain the quota throughout the year. This may explain why the numbers of cod discarded ≥MLS begin to increase after approximately 50% of the quota is fished up. A previous study found that over-quota discarding occurred towards the end of the year [37]. However, if other management regulations restricting the landing of a species are in force, such as a ration system, discarding of individuals ≥MLS may take place earlier in the year when these become restrictive. Regulatory discards <MLS are however controllable to a degree, based on factors such as mesh size, area fished and others influencing their magnitude [30].

In areas and/or periods when the abundance of individuals between minimum retention length and MLS is high, discards will subsequently be high. It would be beneficial to introduce management regulations to restrict the catching of a species until these individuals have reached a length ≥MLS. The obligation for vessels to move fishing ground, real time closures and areas closures are potential measures that could achieve this.

Total catch weight of cod and plaice was found to be positively correlated with discard numbers while a negative correlation was found for Norway lobster. For cod discards <MLS a positive trend was evident up to around 1000 kg where it appears that some sort of saturation point is reached. Similar trends have been found in selectivity trials where L50 increases as catch weight increases beyond a certain threshold [38]. This could be attributed to the meshes directly in front of the catch becoming stretched open, resulting in better selective properties for smaller individuals. This is where gear selectivity has the potential to improve, through the development of gears that provide more stable selectivity. A similar trend was not observed for plaice. It is possible that selectivity does not improve for plaice, and potentially other flatfish species, as catches increase, due to their morphology. Flatfish morphology likely fits better to a relatively closed diamond mesh. Size selectivity of Norway lobster is somewhat more difficult to achieve as it is largely dependent on the way the individuals come in contact with the meshes [39]. However, square meshes have been found to improve the selectivity of Norway lobster [40]. Codends with multiple escape areas having different mesh shapes seems to be a way to improve selectivity of both species [40].

The mismatch between gear selectivity and minimum landing size is a significant contributor to discards, especially for those <MLS [17], [18]. In the Kattegat the minimum mesh size was increased from 70 mm to 90 mm in 2005. While this change was substantial, the 90 mm mesh size still results in high levels of discarding. Selectivity can also be affected by other factors than mesh size, for example twine thickness [17], which are not recorded in discard data. An additional increase in the mesh size would further reduce discards while also causing a loss of other commercially important species that are relatively small, namely sole and Norway lobster. This may suggest that benefits for both reducing discard rates and maintaining valuable catches could be derived from the use of efficient species selective devices rather than only mesh size regulations. Recent experiments conducted in the Kattegat suggest that escape windows can be made more efficient in releasing cod [41], and grids can, in general, be used to reject fish bycatch in directed Norway lobster fisheries [18], [42].

Seasonal discarding was also found to be an influential factor and can be attributed to the targeting behaviour of the fishermen and the condition/behaviour of species during different seasons. For example, it is observed that plaice ≥MLS are discarded more during the first quarter of the year. This can be attributed to the physical condition of plaice throughout the year. In winter and early spring large plaice are of low condition and watery flesh, resulting in lower market value [37]. Therefore, low-value individuals caught at the beginning of the year will be discarded to save quota for higher valued individuals caught at the end of the year [37]. Avoiding the capture of plaice during the winter months when they are of poor physical condition could reduce the number of plaice ≥MLS being discarded. Norway lobster < and ≥MLS are discarded more during the summer when they are targeted the most, while cod in the Kattegat have traditionally been targeted during the first months of the year when higher densities occur due to spawning [43]. High discarding of cod ≥MLS is also observed when quota utilisation is low. This could be due to the targeting behaviour of the fishermen during the first quarter of the year and subsequently discarding more.

The spatial distribution of discards for the three species observed here were all different from one another. Therefore, when considering new management measures to reduce discards, the spatial distribution of discards, especially those <MLS, also needs to be considered. Spatial management can provide a useful tool in protecting juvenile fish by reducing discard rates and can serve as a buffer against management errors and recruitment failure [3]. The most consistent benefit from spatial management, however, is that it provides the necessary economic incentive for fishermen to adopt selective fishing techniques that allow them conditional access to otherwise closed areas [3]. Our findings show that the spatial and temporal variability in the discard rates can potentially be exploited in a general strategy to reduce discards. A similar approach was proposed for the USA mixed species otter trawl fisheries of the Georges Bank-Southern New England region [30]. By limiting directed fishing to times and places where resources are segregated, the quantity of unintended catch could potentially be reduced [30].

A discard ban, which has been proposed for EU fisheries as a major change to the CFP, may result in spatio-temporal improvements to the exploitation of the stocks. The capture and subsequent retention of smaller individuals, as would likely be the case under a discard ban, has the potential to reduce economic revenue to the fishermen, depending on how the quotas are restructured. Therefore, under a discard ban, the issue of discarding becomes less of a concern and a set of new issues emerge, such as minimisation of the initial capture of juveniles that would rapidly fill fishing quotas, enforcement, and alterations in the ecosystem functioning, particularly on the sea bird [13] and benthic scavengers [44] that feed on discards at the surface and at the bottom respectively. If implemented correctly, a discard ban should create economic incentives for the industry to reduce the capture of smaller individuals through improvements in gear selectivity and the spatio-temporal distribution of the fishery. Moreover, it would also improve the reliability of scientific stock assessments by removing the current uncertainty associated with the estimation of discards. However, a discard ban also has the potential to encourage misreporting if not properly enforced. Discard bans have proven to be successful outside the EU (i.e., the demersal fishery in Norway, [45]), and its implementation within the EU fisheries will be dependent on understanding and compliance from the industry.

In our study of the Danish demersal trawl fishery it is evident that discards, and their subcomponents, are affected by a multitude of factors that differ depending on what species/subcomponent is being analysed. The same is valid for different fleets, gears, and areas. The factors that have been shown to influence the discard rates of cod, plaice and Norway lobster are highly species-specific and may not hold for other species. Therefore, extending this type of analysis to other discarded species is necessary to explain the overall discard behaviour in a fishery.

## Supporting Information

Figure S1 **Boxplots of the significant categorical variables of the generalised additive models.**
(TIF)

Table S1 Summary of all models fitted together with their significant variables, GCV scores, and the deviance explained by the models. mesh = mesh size category, JuvAb = Juvenile abundance, catch = Total catch weight, lon = longitude, lat = latitude, quarter = quarter of the year, vessel kW = vessel power, depth = mean fishing depth, vessel = individual vessel id, haul dur = haul duration, yr = year, quota = species quota utilisation.
(XLS)
