# Supplementary figures and images for: Fishery Discards: Factors Affecting Their Variability within a Demersal Trawl Fishery
Source: PLoS One. 2012 Apr 30;7(4):e36409. doi: 10.1371/journal.pone.0036409 (PMC3340337; doi:10.1371/journal.pone.0036409)

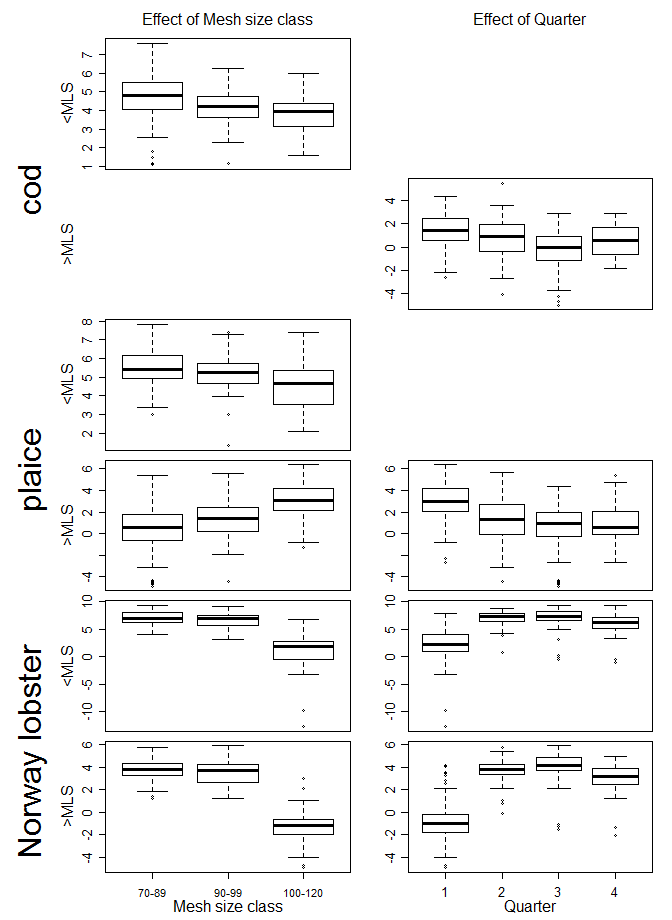

Supplement: Figure S1 — Boxplots of the significant categorical variables of the generalised additive models. (TIF) [file pone.0036409.s001.tif]
